# Associations of inflammatory markers with impaired left ventricular diastolic and systolic function in collagen-induced arthritis

**DOI:** 10.1371/journal.pone.0230657

**Published:** 2020-03-24

**Authors:** Lebogang Mokotedi, Frederic S. Michel, Conrad Mogane, Monica Gomes, Angela J. Woodiwiss, Gavin R. Norton, Aletta M. E. Millen

**Affiliations:** Cardiovascular Pathophysiology and Genomics Research Unit, School of Physiology, Faculty of Health Sciences, University of the Witwatersrand, Johannesburg, South Africa; Faculty of Medical Science - State University of Campinas, BRAZIL

## Abstract

**Background:**

High-grade inflammation may play a pivotal role in the pathogenesis of left ventricular (LV) dysfunction. Evidence to support a role of systemic inflammation in mediating impaired LV function in experimental models of rheumatoid arthritis (RA) remains limited. The aim of the present study was to determine the effects of high-grade systemic inflammation on LV diastolic and systolic function in collagen-induced arthritis (CIA).

**Methods:**

To induce CIA, bovine type-II collagen emulsified in incomplete Freund’s adjuvant was injected at the base of the tail into 21 three-month old Sprague Dawley rats. Nine-weeks after the first immunisation, LV function was assessed by pulsed Doppler, tissue Doppler imaging and Speckle tracking echocardiography. Cardiac collagen content was determined by picrosirius red staining; circulating inflammatory markers were measured using ELISA.

**Results:**

Compared to controls (n = 12), CIA rats had reduced myocardial relaxation as indexed by lateral e’ (early diastolic mitral annular velocity) and e’/a’ (early-to-late diastolic mitral annular velocity) and increased filling pressures as indexed by E/e’. No differences in ejection fraction and LV endocardial fractional shortening between the groups were recorded. LV global radial and circumferential strain and strain rate were reduced in CIA rats compared to controls. Higher concentrations of circulating inflammatory markers were associated with reduced lateral e’, e’/a’, radial and circumferential strain and strain rate. Greater collagen content was associated with increased concentrations of circulating inflammatory markers and E/e’.

**Conclusion:**

High-grade inflammation is associated with impaired LV diastolic function and greater myocardial deformation independent of haemodynamic load in CIA rats.

## Introduction

Heart failure with a preserved ejection fraction (HFpEF) accounts for more than 50% of all heart failure cases and is associated with an increased morbidity and mortality [1]. There are currently no effective treatment strategies for HFpEF [2], which highlights the need for a better understanding of its pathophysiology. Impaired left ventricular (LV) diastolic function has been proposed as a pre-clinical measure that underlies the pathophysiology of, and frequently progresses to HFpEF [3]. The multiple aetiologies of diastolic dysfunction and the heterogeneity of HFpEF [4, 5] underscore the need for further investigations.

Hypertension, diabetes mellitus and obesity comprise traditional cardiovascular risk factors that are associated with diastolic dysfunction [6]. However, these metabolic abnormalities fail to account for all changes in diastolic function [7]. A systemic pro-inflammatory state has been proposed as a mediator for the causal molecular or biochemical mechanisms of diastolic dysfunction in persons exposed to metabolic risk factors [8]. In this regard, diseases that are associated with chronic high-grade inflammation including rheumatoid arthritis (RA), markedly exacerbate the risk for developing diastolic dysfunction and HFpEF compared to the general population [9, 10]. In cross-sectional studies, inflammatory markers including interleukin 6 (IL-6) [11] and tumour necrosis factor-α (TNF-α) [12] levels were independently associated with impaired diastolic function in RA. Although there is some controversy, treatment with biological disease modifying anti-rheumatic agents aimed at reducing inflammation has shown improvements in cardiac function in patients with RA [13]. However, there is currently limited evidence to support a role of inflammation in impaired diastolic function in experimental models of RA.

Chronic systemic inflammation is also associated with heart failure with a reduced ejection fraction (HFrEF) in the general population [14]. Higher levels of inflammatory cytokines adversely affect systolic function and heart failure severity [14], and independently predict mortality in HFrEF [15]. In cross-sectional RA studies, inflammation was not related to reduced ejection fraction [16, 17]. Recently, velocity, displacement, and deformation imaging (strain and strain-rate) as estimated by speckle-tracking echocardiography (STE) were documented to represent valuable tools in the comprehensive and reliable assessment of myocardial systolic function [18–21]. Interestingly, impaired myocardial deformation in the presence of a normal ejection fraction was reported in RA [22–24]. Myocardial deformation is related to inflammation [25, 26], disease activity and/or severity in RA [17, 23, 24]. Until recently, the lack of high-sensitive imaging has limited the use of STE in small animal models. Hence, the effects of high-grade inflammation on STE estimated myocardial function have not been studied in experimental models of RA. In the present study, we assessed the effects of high-grade inflammation on diastolic and systolic function and myocardial deformation and motion as estimated by pulsed and tissue Doppler indices, M-mode echocardiography and STE, respectively, in collagen-induced arthritis (CIA).

## Methods

### Animals and experimental design

All experimental procedures were performed in accordance with the Guide for the Care and Use of Laboratory Animals, Eighth Edition, updated by the US National Research Council Committee in 2011 and were approved by the Animal Ethics Screening Committee (AESC) of the University of the Witwatersrand (AESC number: 2017/03/21C). Thirty-three, three-month-old male Sprague Dawley rats (480-510g) were studied. Rats were housed individually in cages in a temperature-controlled room with a 12-hour light-dark cycle and allowed free access to food and water. During the two week acclimatisation period, blood pressure (BP) was measured twice a week and body weight, paw thickness and articular index scores were measured once a week. Following acclimatisation, rats were randomly assigned to the control group (CNTRL, n = 12) that had no intervention, or the collagen induced arthritis group (CIA, n = 21) that were exposed to high-grade inflammation. Body weight, paw thickness, articular index scores and BP were measured once a week for nine weeks. Echocardiography was performed and blood samples were obtained at the end of the nine week study period.

### Arthritis induction and assessment

Experimental arthritis was induced in rats as previously described [27]. Briefly, bovine type II collagen (Chondrex cat. #20021, Redmond, WA, USA) was dissolved in 0.05M acetic acid by gently stirring overnight at 4°C. Equal amounts of dissolved bovine type II collagen (2mg/ml) and incomplete Freund’s adjuvant (Chondrex cat. #7002, Redmond, WA, USA) were mixed using an electric homogenizer. The arthritis-inducing emulsion was prepared immediately before immunisation. Under general anaesthesia, rats were immunised with 0.2ml (200μg) of the emulsion by a subcutaneous injection at the base of the tail. To ensure a high incidence and severity of arthritis, a 0.1ml (100μg) booster injection was administered seven days after the first immunisation. Control rats received a subcutaneous injection of 0.1ml (100 μg) of 0.05M acetic acid at the base of the tail. To quantitatively evaluate the severity of arthritis, rat paws were scored using a previously described five-point scoring system [28] where 0 = no swelling or focal redness (normal); 1 = slight swelling and/or focal redness; 2 = low-to-moderate oedema, 3 = pronounced oedema with reduced paw function; 4 = excessive oedema with deformity and joint rigidity. The cumulative score for the two hind paws of each rat (maximum score of 8) were used to represent the overall disease severity. As the rat paw arthritis score only provides a subjective quantification of inflammation, hind paw thickness at the ankle and tarsometatarsal joints were measured once every week using a digital calliper as another measure of arthritis severity.

### Non-invasive blood pressure measurements

Blood pressure was measured weekly using the tail-cuff technique (Biopac Systems, Santa Barbara, CA, USA). Each rat was placed in a restrainer with a cuff attached to a heated tail. Measurements were taken at midday to avoid diurnal variation.

### Echocardiography

Nine weeks after the first immunisation, rats were anaesthetised with an intraperitoneal injection of ketamine (100mg.kg^-1^) and xylazine (5mg.kg^-1^). Echocardiography was performed by an experienced observer, according to the American Society of Echocardiography conventions [29] with the rat in the left lateral decubitus position using a high resolution ultrasound probe (10 MHz) coupled to an echocardiogram (Siemens, Acuson SC2000, Diagnostic ultrasound system; Siemens Medical Solutions, USA, Inc.). LV dimensions were determined using two-dimensional directed M-mode echocardiography in the parasternal long axis view during three consecutive beats. LV end systolic (LVESD) and end diastolic (LVEDD) internal diameters and septal (IVST) and posterior wall thickness (PWT) were measured in systole and diastole. Relative wall thickness (RWT) was calculated as (IVST + PWT in diastole)/LVEDD [30].

LV diastolic function was determined from the mitral valve inflow patterns using pulsed Doppler imaging. In the apical 4-chamber view, the early (E) and late (A) diastolic inflow velocity were obtained with the sample volume placed at the mitral valve leaflet tip and expressed as E/A as a marker of relaxation. To determine diastolic function using tissue Doppler imaging (TDI), peak myocardial tissue lengthening velocities during early (e’) and late (a’) diastole were recorded at the lateral mitral annulus in the apical four-chamber view. Data were expressed as e’ (an index of myocardial relaxation), e’/a’ (an index of myocardial stiffness) and E/e’ (an index of LV filling pressure).

LV pump and myocardial systolic function was determined by calculating LV ejection fraction (EF) using the Teichholz method [30] and LV endocardial fractional shortening (FSend) using the equation LV (EDD-ESD)/EDD [30], respectively. To further evaluate systolic function Speckle tracking Doppler was used to obtain B-mode images to determine LV strain, strain rate, velocity and displacement in the parasternal short axis view (circumferential or rotational). B-mode video loops were selected based upon image quality and well-defined endocardial and epicardial borders with no substantial image artefacts. An average of at least five consecutive heartbeats was used to minimize beat-to-beat variability for all measurements. The endocardium and epicardium were traced semi-automatically using vendor’s software. The traces were manually adjusted to ensure adequate tracking of the endocardial and epicardial borders. Tracked images were processed in a frame-by-frame manner for strain measurements. Strain, strain rate, velocity and displacement were calculated in the radial and circumferential planes. Segmental analyses were performed on the short-axis images with the left ventricle being split into the following regions: anterior septal, anterior, lateral, posterior, inferior and septal regions. The average global strain values were obtained from six independent anatomical segments of the left ventricle.

### Serum concentrations of inflammatory markers

Rats were sacrificed with an intraperitoneal injection of ketamine 200 mg.kg^-1^ and xylazine 10 mg.kg^-1^ followed by thoracotomy. After thoracotomy, blood was sampled and allowed to clot for 2 hours at room temperature. Blood was centrifuged and serum was collected and stored at -80°C until assayed. Serum concentrations of tumor necrosis factor alpha (TNF-α), interleukin 6 (IL-6), interleukin 1β (IL-1β) and C-reactive protein (CRP) were measured by ELISA using commercially available ELISA kits according to the instructions of the manufacturer (Elabscience Biotechnology Co. Ltd, Wuhan, China). Each sample was measured in duplicate. The lower detection limit for TNF-α, IL-6, IL-1β and CRP were 78.13 pg/ml, 62.50 pg/ml, 31.2 pg/ml and 0.31 ng/ml respectively, all with coefficients of variation of <10%.

### Total collagen content

Cardiac tissue samples were fixed in 10% buffered formalin and routinely processed for paraffin embedding. Five μm thick tissue sections were deparaffinised, rehydrated and stained with a 0.1% Sirius Red solution dissolved in aqueous saturated picric acid for 60 min at room temperature. After washing in acidified water, slides were dehydrated and mounted with DPX mounting. The tissue sections were analysed using a Zeiss Axioskop 2 Plus microscope equipped with a Zeiss AxioCam (Zeiss, Peabody, MA, USA). Tissue sections viewed under bright-field and polarized light were obtained with a 10x objective lens (x100 magnification). Using ImageJ software, the collagen area fraction was calculated for each tissue section by dividing the collagen area by the total tissue area.

### Statistical analysis

Data are expressed as means ± SD. Data analysis was performed using SAS software version 9.4 (SAS Institute Inc., Cary, North Carolina, USA). Unpaired, two-tailed t-tests were performed to determine differences in echocardiographic measures and inflammatory markers between the CIA and control groups. Associations between diastolic and systolic function parameters and inflammatory markers were determined by Pearson’s correlation coefficients. A P value < 0.05 was considered statistically significant.

## Results

### Characterization of experimental model

**Fig 1:**
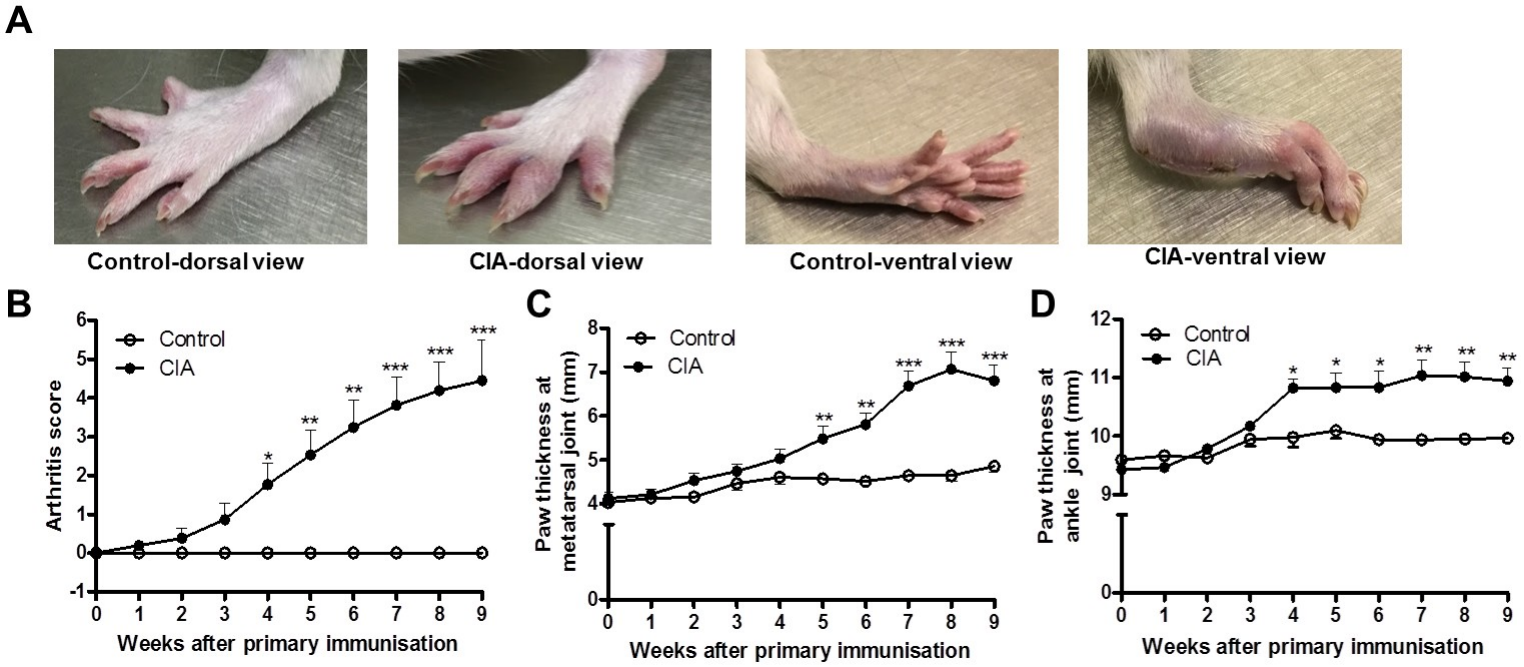
Macroscopic observation of joint swelling in collagen-induced arthritis (CIA) rats. (A) Photographs of hind paws, (B) arthritis scores, (C) paw thickness at the tarsometatarsal joint and (D) paw thickness at the ankle joint in control and CIA rats nine weeks after the primary immunisation. Open circles represent the control group (n = 12) and closed circles represent the CIA (n = 21) group (unpaired t-test). Data presented as mean ± SD. *P < 0.05 versus control group; **P < 0.01 versus control group; ***P < 0.0001 versus control group.

The onset of arthritis in the CIA group occurred within 21–28 days after the first immunisation. Clear signs of inflammation including erythema, oedema and joint rigidity were observed in the CIA group (Fig 1A). Compared to the control group, the CIA group showed a significantly higher arthritis score (Fig 1B) four weeks after immunisation that persisted for the duration of the study. CIA rats also showed increased swelling at the tarsometatarsal joint (Fig 1C) from week five to week nine and increased swelling at the ankle joint (Fig 1D) from week four until week nine after the primary immunisation.

### Body weight, blood pressure and inflammatory markers

Table 1 shows the body weight, blood pressure and inflammatory markers of the control and CIA groups. Nine weeks after the primary immunisation, there were no significant differences in body weight, systolic or diastolic blood pressure between the groups (all p>0.05). Serum concentrations of TNF-α, IL-1β, IL-6 and CRP were significantly higher in the CIA group compared to the control group (all p<0.0001).

**Table 1:** Body weights, tail cuff blood pressure and inflammatory markers in controls and collagen induced arthritis rats.

	Control(n = 12)	CIA(n = 21)	P
Sample (n)	12	21	
Body weight (g)	549 ± 56	529 ± 57	0.35
Blood pressure			
Systolic blood pressure (mm Hg)	133 ± 3	137 ± 13	0.40
Diastolic blood pressure (mm Hg)	92 ± 5	94 ± 6	0.38
Inflammatory markers			
Tumor necrosis factor-α (pg/ml)	277.9 ± 88.1	645.9 ± 128.8	<0.0001
Interleukin-6 (pg/ml)	100.4 ± 72.9	377.8 ± 92.6	<0.0001
Interleukin-1β (pg/ml)	104.5 ± 55.4	245.5 ± 51.3	<0.0001
C-reactive protein (ng/ml)	0.2 ± 0.3	1.0 ± 0.4	<0.0001

Data expressed as means ± SD. CIA, collagen induced arthritis

### Cardiac weight and geometry

Table 2 shows the cardiac geometry of the control and CIA groups. Although the control group had similar heart weights as the CIA group (p>0.05) at termination, the (mean ± SD) heart weight indexed to body weight (control: 2.42 ± 0.20; CIA: 2.63 ± 0.26, p = 0.02), LV weight (control: 0.95 ± 0.09 g; CIA: 1.03 ± 0.12 g, p = 0.04) and LV weight/ indexed to body weight (control: 1.74 ± 0.12; CIA: 1.97 ± 0.26, p = 0.01) were significantly higher in the CIA group compared to the control group. The posterior wall thickness in systole (mean ± SD; control: 2.00 ± 0.02 mm; CIA: 2.30 ± 0.03 mm, p = 0.03) and diastole (mean ± SD; control: 2.90 ± 0.03 mm; CIA: 3.14 ± 0.03 mm, p = 0.002) were higher in the CIA group compared to controls, which resulted in an increased relative wall thickness in the CIA group (mean ± SD; control: 0.59 ± 0.10 mm; CIA: 0.69 ± 0.09 mm, p = 0.01).

**Table 2:** Left ventricular geometry, diastolic and systolic function in controls and collagen induced arthritis rats.

	Control (n = 12)	CIA (n = 21)	P
Cardiac geometry			
Heart weight (g)	1.32 ± 0.10	1.38 ± 0.13	0.17
Heart weight/body weight x 10^3^	2.42 ± 0.20	2.63 ± 0.26	0.02
Right ventricular weight (g)	0.28 ± 0.05	0.27 ± 0.06	0.38
LV weight (g)	0.95 ± 0.09	1.03 ± 0.12	0.04
LV weight/body weight x 10^3^	1.74 ± 0.12	1.97 ± 0.26	0.01
LV end diastolic diameter (mm)	6.95 ± 0.07	6.65 ± 0.07	0.25
LV end diastolic posterior wall thickness (mm)	2.00 ± 0.02	2.30 ± 0.03	0.002
LV end systolic diameter (mm)	4.15 ± 0.05	3.90 ± 0.07	0.28
LV end systolic posterior wall thickness (mm)	2.90 ± 0.03	3.14 ± 0.03	0.03
Relative wall thickness (mm)	0.59 ± 0.10	0.69 ± 0.09	0.01
Left ventricular diastolic function			
E (cm/s)	133 ± 17	134 ± 15	0.83
E/A	1.95 ± 0.35	1.75 ± 0.29	0.14
e’ (cm/s)	4.51 ± 0.81	3.45 ± 0.39	<0.0001
e’/a’	1.42 ± 0.22	1.04 ± 0.26	0.0002
E/e’	31.78 ± 10.42	39.45 ± 7.34	0.04
Left ventricular systolic function			
Stroke volume (ml)	0.59 ± 0.19	0.53 ± 0.15	0.31
Ejection fraction (%)	75.71 ± 7.75	76.95 ± 9.61	0.71
Endocardial fractional shortening (%)	40.07 ± 6.41	41.52 ± 8.56	0.60
Global radial strain (%)	11.82 ± 1.02	9.68 ± 1.15	<0.0001
Global circumferential strain (%)	-26.40 ± 1.38	-21.86 ± 4.74	0.01
Global radial strain rate (1/s)	2.10 ± 0.41	1.71 ± 0.19	0.003
Global circumferential strain rate (1/s)	-3.61 ± 0.47	-3.10 ± 0.20	0.001
Global radial velocity (cm/s)	1.37 ± 0.25	1.09 ± 0.25	0.01
Global circumferential velocity (degree/s)	54.47 ± 9.05	44.66 ± 8.59	0.01
Global radial displacement (mm)	0.70 ± 0.15	0.66 ± 0.17	0.55
Global circumferential displacement (degree)	1.28 ± 0.25	1.10 ± 0.31	0.16

Data expressed as means ± SD. CIA, collagen induced arthritis; LV, left ventricular

### Left ventricular diastolic function

Table 2 shows that compared to the control group, the CIA group had reduced lateral e’ (mean ± SD; control: 4.51 ± 0.81 cm/s; CIA: 3.45 ± 0.39 cm/s, p<0.0001) and e’/a’ (mean ± SD; control: 1.42 ± 0.22; CIA: 1.04 ± 0.26, p = 0.0002) and a higher E/e’ (mean ± SD; control: 31.78 ± 10.42; CIA: 39.45 ± 7.34, p = 0.04). There were no significant differences in E or E/A between the groups (both p>0.05).

### Left ventricular systolic function

There were no differences in stroke volume (p = 0.31), ejection fraction (p = 0.71) or endocardial fractional shortening (p = 0.60) between the groups (Table 2). Global radial strain (mean ± SD; control: 11.82 ± 1.02%; CIA: 9.68 ± 1.15%, p<0.0001), global circumferential strain (mean ± SD; control: -26.40 ± 1.38%; CIA: -21.86 ± 4.74%, p = 0.01), global radial strain rate (mean ± SD; control: 2.10 ± 0.41 1/s; CIA: 1.71 ± 0.19 1/s, p = 0.003) and global circumferential strain rate (mean ± SD; control: -3.61 ± 0.47 1/s; CIA: -3.10 ± 0.20 1/s, p = 0.001) were significantly reduced in the CIA group compared to the control group (Table 2). Global radial velocity (mean ± SD; control: 1.37 ± 0.25 cm/s; CIA: 1.09 ± 0.25 cm/s, p = 0.01) and global rotational velocity (mean ± SD; control: 54.47 ± 9.05 degree/s; CIA: 44.66 ± 8.59 degree/s, p = 0.01) were significantly reduced in the CIA group compared to the control group, however global radial displacement and global circumferential displacement were similar between the groups (both p>0.05; Table 2).

In segmental analysis in the short-axis radial strain, circumferential strain, radial strain rate and circumferential strain rate were predominantly impaired at the anterior-septal, anterior and septal LV segments in the CIA group compared to controls (all p<0.05; S1 Table). In the lateral LV segment, only circumferential strain (p = 0.02) and radial strain rate (p = 0.05) were reduced in the in the CIA group compared to controls (S1 Table). Strain or strain rate were not different between the groups in the posterior or inferior segments (all p>0.05; S1 Table). S2 Table shows that radial velocity was significantly reduced at the anterior septal (p = 0.02), anterior (p = 0.05) and septal (p = 0.03) LV segments in the CIA group compared to controls. Rotational velocity was significantly reduced at the anterior septal (p = 0.004) and anterior (p = 0.04) LV segments. There were no differences in the radial or circumferential displacement in any of the segments between the groups (S2 Table).

### Total collagen content

**Fig 2:**
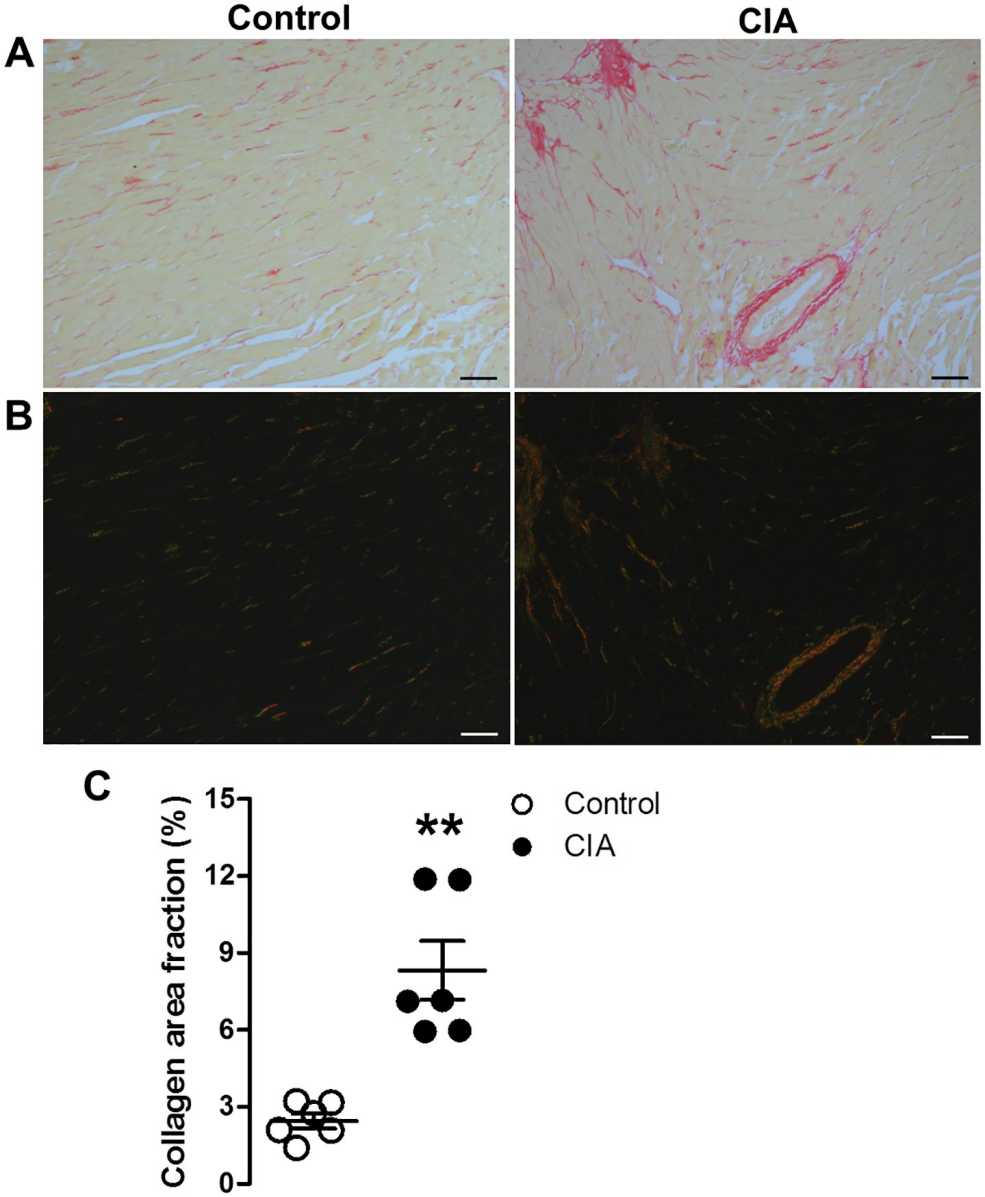
Total collagen content in cardiac tissue of control and collagen-induced arthritis (CIA) rats. Representative Picrosirius red stained micrographs imaged at x100 magnification viewed in (a) bright-field and under (b) polarized light. (c) Total collagen content (% area fraction) calculated from Picrosirius red stained sections. Data presented as mean ± SD; unpaired t-test, **P < 0.05 versus control group; n = 6 per group. Scale bar = 20μm.

Fig 2A shows greater collagen accumulation in CIA rats compared to controls as evidenced by the increased red staining of cardiac tissue sections visualized under bright-filed microscopy. Fig 2B shows cardiac tissue sections under polarized light, where large collagen fibres appear orange or yellow and thin fibres appear green. The total collagen content was significantly greater in the CIA group compared to controls (mean ± SD; control: 2.45 ± 0.71%; CIA: 8.62 ± 3.39%, p = 0.01; Fig 2C).

### Associations between inflammatory markers, cardiac geometry and collagen content

**Fig 3:**
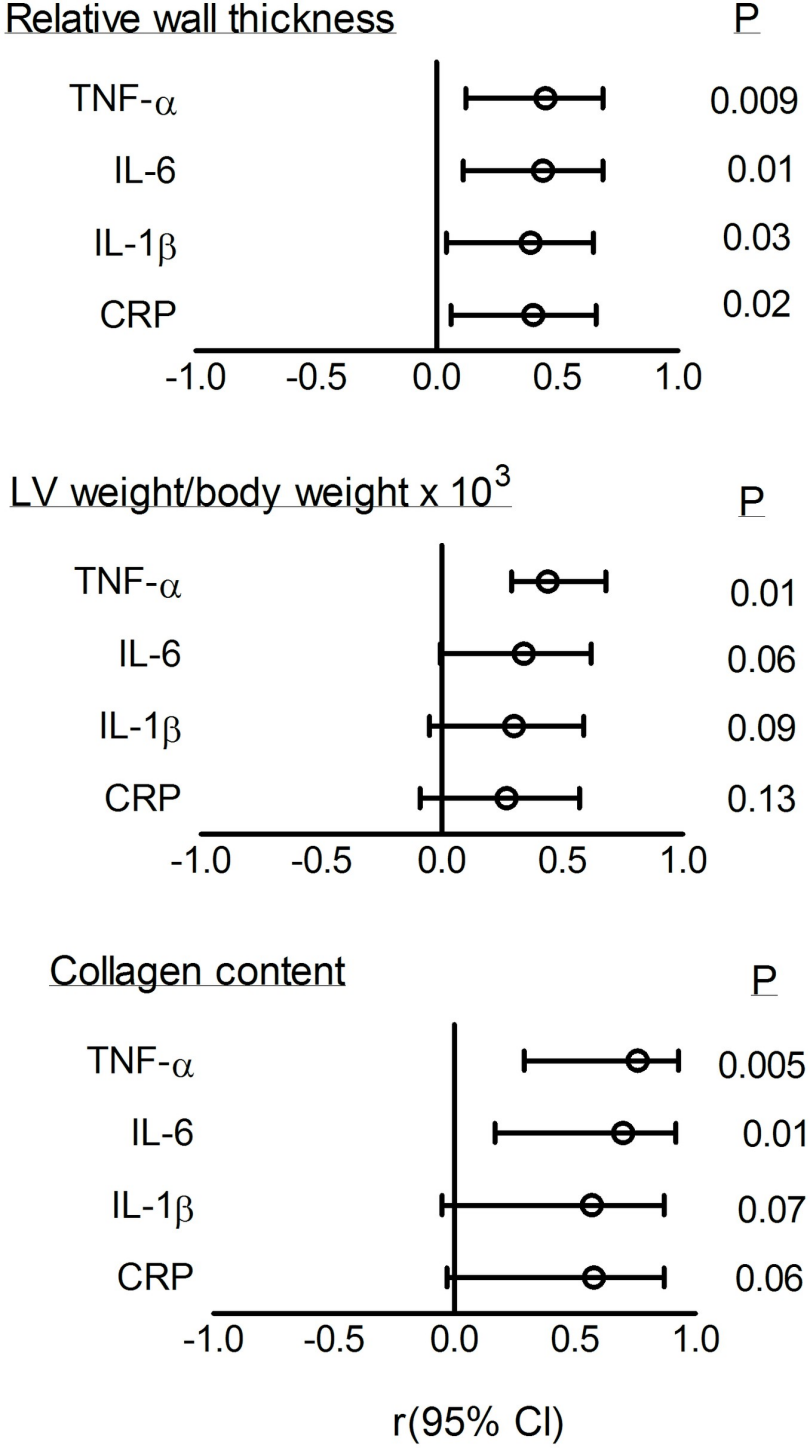
Associations of cardiac geometry and total collagen content with inflammatory cytokines. TNF-α, tumor necrosis factor alpha; IL-6, interleukin 6; IL-1β, interleukin 1 beta; CRP, C-reactive protein. Open circles represent the correlation coefficient (r) and horizontal lines represent the 95% confidence intervals (Cl) (Pearson’s correlation).

Fig 3 shows that relative wall thickness was related to increased circulating inflammatory marker concentrations (TNF-α: r = 0.45; p = 0.009, IL6: r = 0.44; p = 0.01, IL-1β: r = 0.39; p = 0.03, CRP: r = 0.40; p = 0.02). LV weight indexed to body weight was related to increased TNF-α (r = 0.44; p = 0.01) and there was a trend toward significance with IL-6 (r = 0.34; p = 0.06). Increased collagen content was associated with higher serum concentrations of TNF-α (r = 0.76; p = 0.005) and IL-6 (r = 0.70; p = 0.01) and there was a trend towards significance with IL-1β (r = 0.57; p = 0.07) and CRP (r = 0.58; p = 0.06).

### Associations between inflammatory markers and diastolic function

**Fig 4:**
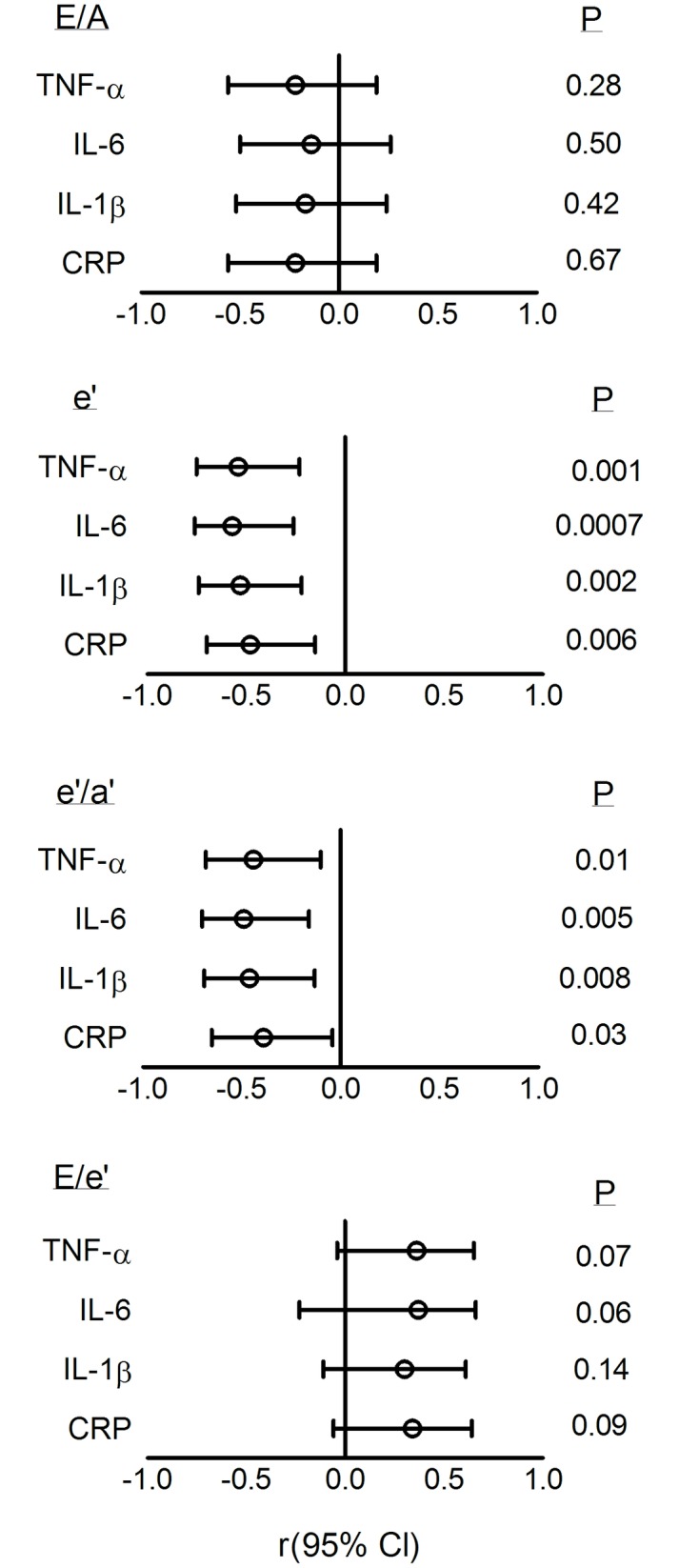
Associations between left ventricular diastolic function markers and circulating inflammatory markers. TNF-α, tumor necrosis factor alpha; IL-6, interleukin 6; IL-1β, interleukin 1 beta; CRP, C-reactive protein. Open circles represent the correlation coefficient (r) and horizontal lines represent the 95% confidence intervals (Cl) (Pearson’s correlation).

Fig 4 shows the associations between circulating inflammatory marker concentrations and markers of diastolic function. Reduced e’ was associated with higher serum concentrations of TNF-α (r = -0.54; p = 0.001), IL-6 (r = -0.57; p = 0.0007), IL-1β (r = -0.53; p = 0.002) and CRP (r = -0.48; p = 0.006). Reduced e’/a’ was associated with higher serum concentrations of TNF-α (r = -0.44; p = 0.01), IL-6 (r = -0.49; p = 0.005), IL-1β (r = -0.46; p = 0.008) and CRP (r = -0.39; p = 0.03, Fig 4). There was no association between E/A and serum concentrations of TNF-α (r = - 0.22; p = 0.28), IL-6 (r = - 0.14; p = 0.50), IL-1β (r = -0.17; p = 0.42), or CRP (r = -0.09, p = 0.67; Fig 4). There was a trend toward an association between E/e’ and serum concentrations of TNF-α (r = 0.36; p = 0.07), and IL-6 (r = 0.37; p = 0.06) (Fig 4).

### Associations between inflammatory markers and systolic function

There were no significant associations between serum concentrations of inflammatory markers and ejection fraction (TNF-α: r = 0.22; p = 0.22, IL-1β: r = 0.19; p = 0.28, IL-6: r = 0.20; p = 0.26, CRP: r = 0.17; p = 0.37) or endocardial fractional shortening (TNF-α: r = -0.19; p = 0.29, IL-β: r = -0.19; p = 0.30, IL-6: r = -0.14; p = 0.45, CRP: r = -0.08, p = 0.65).

**Fig 5:**
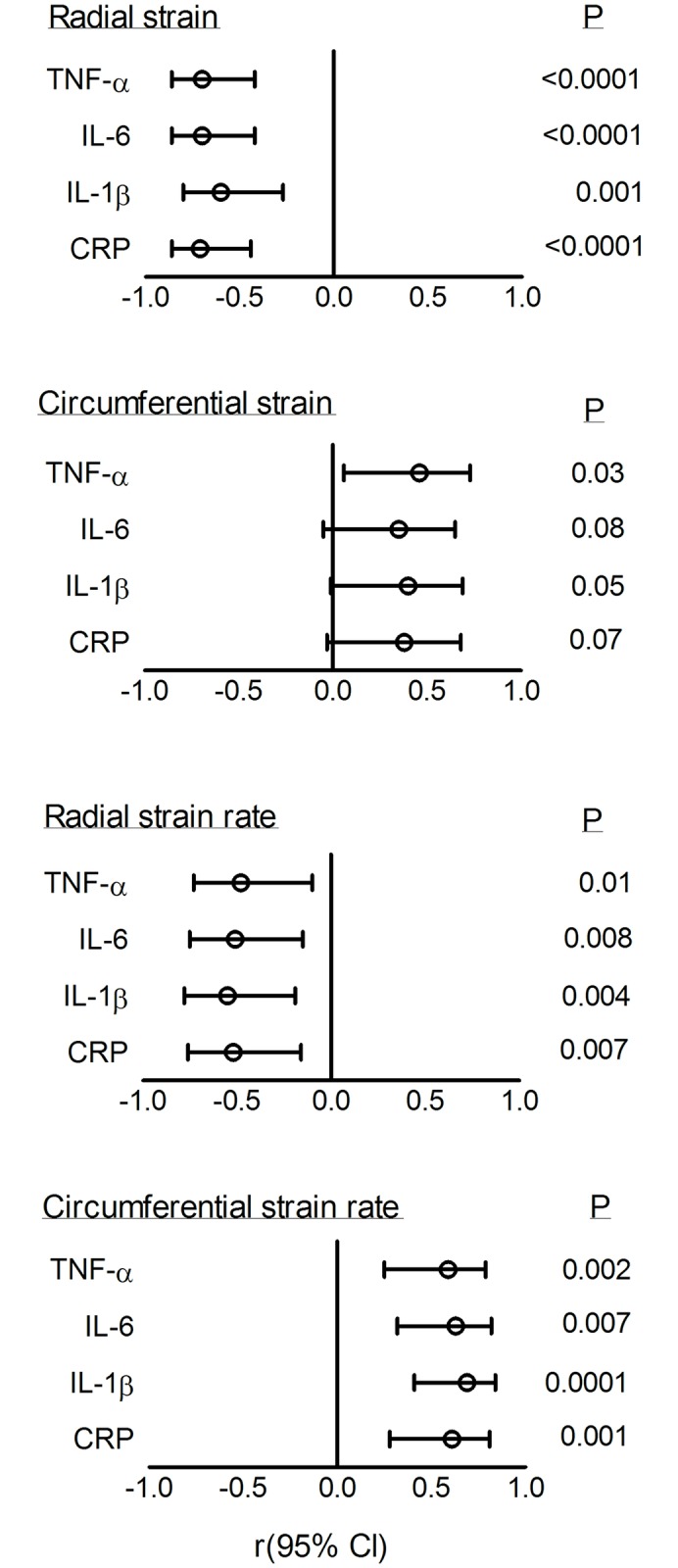
Associations between left ventricular myocardial deformation (strain and strain rate) and circulating inflammatory markers. TNF-α, tumor necrosis factor alpha; IL-6, interleukin 6; IL-1β, interleukin 1 beta; CRP, C-reactive protein. Open circles represent the correlation coefficient (r) and horizontal lines represent the 95% confidence intervals (Cl) (Pearson’s correlation).

Fig 5 shows that greater myocardial deformation was associated with higher levels of circulating inflammatory markers. Reduced radial strain was associated with higher serum concentrations of TNF-α (r = -0.70; p<0.0001), IL-6 (r = -0.70; p<0.0001), IL-1β (r = -0.60; p = 0.001) and CRP (r = -0.71; <0.0001; Fig 5). Reduced (less negative) circumferential strain was associated with higher serum concentrations of TNF-α (r = 0.46; p = 0.03) and IL-1β (r = 0.40; p = 0.05; Fig 5). No significant associations were shown between circumferential strain and IL-6 (r = 0.35; p = 0.08) or CRP (r = 0.38; p = 0.07) concentrations (Fig 5). Reduced radial strain rate was associated with higher TNF-α (r = -0.48; p = 0.01), IL-6 (r = -0.51; p = 0.008), IL-1β (r = -0.55; p = 0.004) and CRP (r = -0.52; p = 0.007) concentrations (Fig 5). Reduced (less negative) circumferential strain rate was associated with higher serum concentrations of TNF-α (r = 0.59; p = 0.002), IL-6 (r = 0.63; p = 0.007), IL-1β (r = 0.69; p = 0.0001) and CRP (r = 0.61; p = 0.001; Fig 5). There were no associations between markers of myocardial motion (velocity and displacement) and circulating inflammatory markers (S1 Fig).

## Discussion

In the present study, CIA in adult, male Sprague Dawley rats caused impaired LV relaxation (decreased e’) and increased LV stiffness (decreased e’/a’) and LV filling pressures (increased E/e’). The diastolic abnormalities were noted despite a normal blood pressure and unaltered mitral inflow patterns (E/A). Although LV chamber pump function (ejection fraction) and contractility (fractional shortening) were preserved, CIA rats demonstrated early myocardial dysfunction as indexed by myocardial deformation (radial and circumferential strain and strain rate). Elevated inflammatory cytokines were associated with impaired diastolic function (e’ and e’/a’), increased collagen content and global radial and circumferential strain and strain rate. The present findings suggest that, independent of load, LV diastolic function and myocardial deformation are adversely affected when exposed to high-grade inflammation in rats.

Previous cross-sectional studies have reported impaired diastolic function in RA patients with adverse inflammatory profiles [10–12, 14]. Accordingly, the present study showed that TDI-determined e’, an index of myocardial relaxation, was reduced in CIA rats compared to controls and was associated with increased circulating inflammatory markers. However, no changes in pulsed Doppler markers of diastolic function (E/A) were observed in CIA rats compared to controls. In contrast, one study showed impaired early diastolic filling velocity (E) in female CIA rats [31]. No other markers of diastolic function were assessed in the respective investigation [31]. The discrepancies may be explained by the onset, severity and duration of the different arthritic protocols used in the latter and current studies.

Besides impaired active relaxation, passive myocardial stiffness contributes to the pathogenesis of diastolic dysfunction [4, 5]. Hypertension has been linked to increased myocardial collagen content, collagen cross linking and myocardial fibrosis which leads to diastolic dysfunction [32–34]. However recent studies indicate that hypertension cannot fully account for the development of diastolic dysfunction in RA patients [7, 11]. Similar to previous studies in the CIA rat model [35, 36], no differences in blood pressure were observed between the CIA and control groups in the current investigation. However, CIA rats have increased ventricular stiffness, as indexed by a reduced e’/a’, and increased collagen LV content, both of which have been associated with increased circulating inflammatory markers. The current study is thus the first to demonstrate associations between inflammatory markers with increased cardiac remodeling (collagen content), impaired myocardial relaxation and increased passive stiffness, independent of load (blood pressure), in an animal model of RA.

Growing evidence suggests that inflammatory cytokines mediate several cellular changes that cause abnormalities in myocardial relaxation. First, inflammation has been shown to impair the active calcium-dependent processes involved in myocardial relaxation [37, 38]. Additionally, inflammatory cytokines mediate hypertrophic remodelling and myocardial fibrosis through regulation of collagen synthesis and matrix metalloproteinase activity of cardiac fibroblasts [39, 40]. Moreover, recent findings support a role for systemic inflammation in the hypophosphorylation of the cytoskeletal protein titin, which contributes to passive myocardial stiffness [41]. Both collagen and titin phosphorylation contribute to passive myocardial stiffness [42]. Previous studies documented that myocardial stiffness [43], collagen content [44] and impaired relaxation [45] each relate to increased LV filling pressures. In the present study, inflammatory markers tended to relate to increased filling pressure without reaching significance (p = 0.06 to 0.14), suggesting that increased filling pressure may not directly relate to inflammation in the CIA rat model and may be explained by the extra-cellular matrix driven passive stiffness and calcium dependent relaxation abnormalities induced by inflammation. Taken together, the present findings support the recent suggestions that inflammation forms an important part of the pathophysiological mechanisms leading to myocardial remodelling and diastolic dysfunction [8]. Future studies have to investigate the cellular and molecular mechanisms involved in the inflammation-induced diastolic abnormalities in CIA rats.

In previous cross-sectional RA studies, inflammatory cytokines have been associated with increased myocardial deformation [22, 23, 25, 26]. Similar to findings in RA patients [22, 23, 25, 26], the present study showed that LV systolic myocardial deformation (radial and circumferential strain and strain rate) and myocardial motion (circumferential rotation rate and radial velocity) were impaired in the CIA group despite a normal pump function and contractility. Myocardial deformation, but not contractility, was associated with increased circulating inflammatory cytokine concentrations. However, the present findings contrast with impaired contractility, as assessed by conventional approaches, in an *in vivo* mouse model of collagen antibody induced arthritis [46]. While the collagen antibody induced arthritis model shares many characteristics with CIA, the differences in onset and severity of inflammation as well as duration of the studies may explain the differences in contractility between these two studies. Although relaxation and contractility are impaired, ejection fraction may be preserved in HFpEF because of the hypertrophied myocardial wall [47]. In addition, concentric cardiac remodelling increases myocardial tissue stress and strain [47]. In the present study, concentric remodelling, as observed by increased relative wall thickness, may have ensured the maintenance of ejection fraction while increasing myocardial strain.

In the present CIA investigation, myocardial deformation and motion were consistently reduced in the anterior septal, anterior and septal LV myocardial segments. The lateral LV segment was affected to a lesser extent whereas the inferior and posterior walls were unaltered. Previous studies have shown that the septal wall has a greater sensitivity to fibrosis [48] and increased septal myocardial stress has been associated with early structural remodelling in patients with HFpEF [49]. To the best of our knowledge, the present study is the first to demonstrate associations between inflammatory markers with early myocardial deformation in an animal model of RA. Although conventional echocardiography and TDI are considered reliable methods in assessing pump function and contractility, these procedures lack sensitivity in detecting subtle myocardial changes [19, 20]. STE may provide a more sensitive alternative to identify early myocardial contractile changes in RA.

The current study presents with limitations. Although associations were demonstrated between circulating inflammatory markers and impaired cardiac function, the direct casual effect of inflammation on impaired cardiac function can only be implied. Future studies have to determine whether inflammation alters the cellular mechanisms responsible for the changes in LV function seen in the current study, and whether immunosuppressive drugs restore the inflammation-induced cardiac impairments. Secondly, non-invasive approaches rather than catheter-based systems were used to assess diastolic function in the present study. However, tissue Doppler indices of diastolic function were previously shown to correlate well with invasively measured LV relaxation, stiffness and filling pressures [42]. Thirdly, the present study focused on short-axis circumferential and radial STE analyses. Although longitudinal STE analyses may have provided additional information on LV myocardial deformation and motion, circumferential STE analyses are highly reliable in the assessment of LV function [21]. Fourthly, circulating CRP levels were measured using a standard ELISA CRP assay kit which may not be sensitive enough to detect lower CRP levels, especially for low concentrations of CRP. Lastly, the effects of CIA on cardiac function were assessed in male rats. Future studies may determine whether inflammation impairs cardiac function in female rats.

In conclusion, the present study showed that high-grade inflammation is associated *in vivo* with impaired LV diastolic function and greater subclinical myocardial deformation, independent of blood pressure. These findings add to the evidence that systemic inflammation may mediate myocardial remodelling leading to diastolic dysfunction and myocardial deformation. Considering the lack of adequate pharmacological therapy for the management of HFpEF, further research should determine the effect of anti-inflammatory substances in the prevention and management of HFpEF.

## Supporting information

S1 Fig Associations between left ventricular myocardial motion (velocity and displacement) and circulating inflammatory markers. TNF-α, tumor necrosis factor alpha; IL-6, interleukin 6; IL-1β, interleukin 1 beta; CRP, C-reactive protein. Open circles represent the correlation coefficient (r) and horizontal lines represent the 95% confidence intervals (Cl) (Pearson’s correlation).
(TIF)

S1 Table Short- axis systolic segmental strain and strain rate in the CIA and control groups. (DOCX)

S2 Table Short- axis systolic segmental velocity and displacement in the CIA and control groups. (DOCX)
