# Supplementary figures and images for: Associations of inflammatory markers with impaired left ventricular diastolic and systolic function in collagen-induced arthritis
Source: PLoS One. 2020 Mar 24;15(3):e0230657. doi: 10.1371/journal.pone.0230657 (PMC7092986; doi:10.1371/journal.pone.0230657)

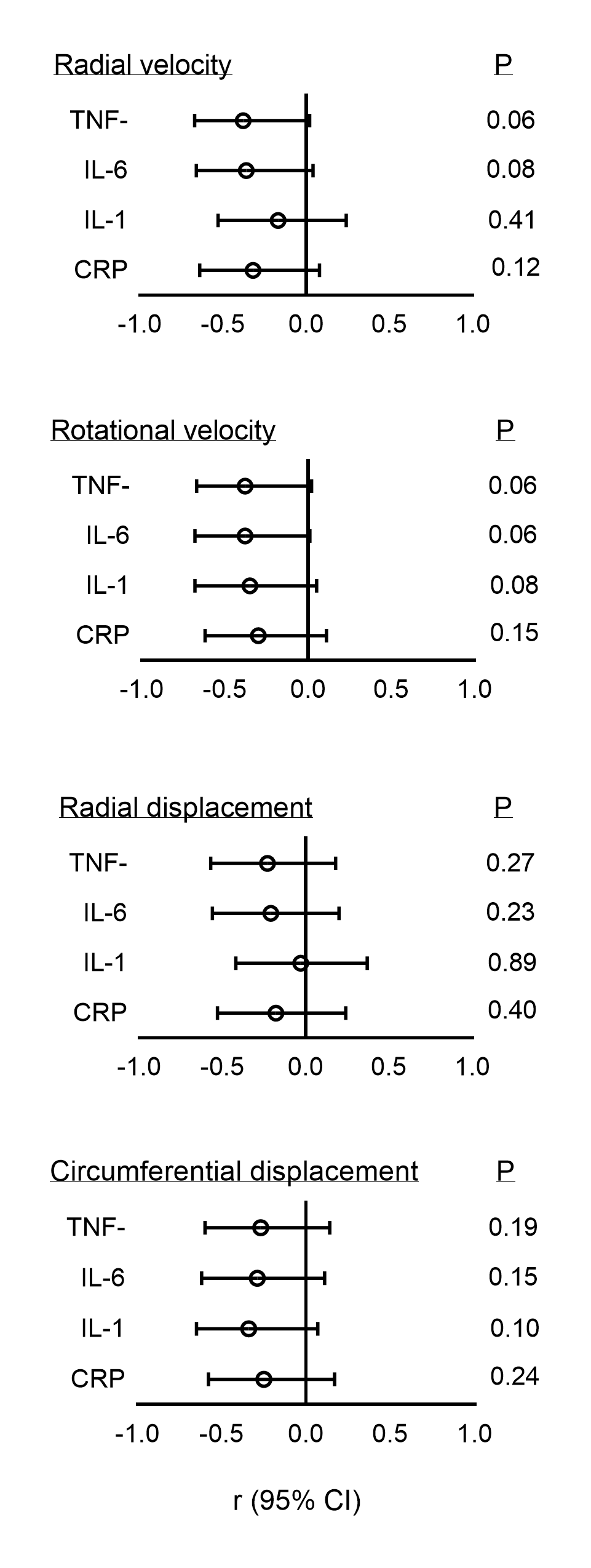

Supplement: S1 Fig — TNF-α, tumor necrosis factor alpha; IL-6, interleukin 6; IL-1β, interleukin 1 beta; CRP, C-reactive protein. Open circles represent the correlation coefficient (r) and horizontal lines represent the 95% confidence intervals (Cl) (Pearson’s correlation). (TIF) [file pone.0230657.s001.tif]
