# Supplementary material for: Associations of inflammatory markers with impaired left ventricular diastolic and systolic function in collagen-induced arthritis
Source: PLoS One. 2020 Mar 24;15(3):e0230657. doi: 10.1371/journal.pone.0230657 (PMC7092986; doi:10.1371/journal.pone.0230657)
Supplement: S1 Table — (DOCX) [file pone.0230657.s002.docx]

**S1 Table. Short- axis systolic segmental strain and strain rate in the CIA and control groups.**

|  | **Control (n=12)** | **CIA (n=21)** | **P** |
| --- | --- | --- | --- |
| **Radial strain (1/s)** |  |  |  |
| Anterior Septal | **14.96 ± 3.13** | **11.61 ± 3.79** | **0.03** |
| Anterior | **15.89 ± 3.09** | **12.06 ± 4.78** | **0.04** |
| Lateral | 13.29 ± 3.78 | 12.28 ± 6.44 | 0.66 |
| Posterior | 13.90 ± 3.79 | 12.77 ± 3.80 | 0.47 |
| Inferior | 12.71 ± 3.49 | 11.29 ± 4.21 | 0.49 |
| Septal | **13.39 ± 6.28** | **8.31 ± 2.83** | **0.01** |
| **Circumferential strain (1/s)** |  |  |  |
| Anterior Septal | **-22.85 ± 7.41** | **-16.95 ± 5.45** | **0.03** |
| Anterior | **-28.04 ± 6.63** | **-21.48 ± 6.66** | **0.02** |
| Lateral | **-27.95 ± 6.24** | **-21.36 ± 7.27** | **0.03** |
| Posterior | -25.99 ± 7.21 | -22.13 ± 8.17 | 0.24 |
| Inferior | -26.37 ± 6.42 | -21.79 ± 7.61 | 0.28 |
| Septal | **-26.68 ± 4.56** | **-22.34 ± 5.09** | **0.04** |
| **Radial strain rate (%)** |  |  |  |
| Anterior Septal | **2.07 ± 0.18** | **1.79 ± 0.48** | **0.05** |
| Anterior | **2.09 ± 0.24** | **1.79 ± 0.35** | **0.03** |
| Lateral | **2.11 ± 0.23** | **1.80 ± 0.42** | **0.05** |
| Posterior | 2.28 ± 0.38 | 2.05 ± 0.63 | 0.32 |
| Inferior | 2.03 ± 0.21 | 1.71± 0.34 | 0.21 |
| Septal | **2.07 ± 0.47** | **1.59 ± 0.49** | **0.02** |
| **Circumferential strain rate (%)** |  |  |  |
| Anterior Septal | **-3.28 ± 0.77** | **-2.47 ± 0.61** | **0.01** |
| Anterior | **-3.59 ± 0.57** | **-3.01 ± 0.68** | **0.05** |
| Lateral | -3.62 ± 0.36 | -3.07 ± 0.81 | 0.06 |
| Posterior | -3.62 ± 0.39 | -3.56 ± 0.96 | 0.87 |
| Inferior | -3.54 ± 0.45 | -3.24 ± 0.72 | 0.61 |
| Septal | **-3.99 ± 0.46** | **-3.16 ± 0.36** | **0.05** |

Data expressed as means ± SD. Significant values are represented in bold. CIA, collagen induced arthritis
