# Supplementary material for: Associations of inflammatory markers with impaired left ventricular diastolic and systolic function in collagen-induced arthritis
Source: PLoS One. 2020 Mar 24;15(3):e0230657. doi: 10.1371/journal.pone.0230657 (PMC7092986; doi:10.1371/journal.pone.0230657)
Supplement: S2 Table — (DOCX) [file pone.0230657.s003.docx]

**S2 Table.** **Short- axis systolic segmental velocity and displacement in the CIA and control groups.**

|  | **Control (n=12)** | **CIA (n=21)** | **P** |
| --- | --- | --- | --- |
| **Radial velocity (cm/s)** |  |  |  |
| Anterior Septal | **1.28 ± 0.24** | **1.08 ± 0.16** | **0.02** |
| Anterior | **1.24 ± 0.28** | **1.03 ± 0.23** | **0.05** |
| Lateral | 1.19 ± 0.23 | 1.04 ± 0.27 | 0.15 |
| Posterior | 1.16 ± 0.23 | 1.12 ± 0.18 | 0.67 |
| Inferior | 1.18 ± 0.26 | 1.14 ± 0.21 | 0.68 |
| Septal | **1.26 ± 0.27** | **1.04 ± 0.21** | **0.03** |
| **Rotational velocity (degree/s)** |  |  |  |
| Anterior Septal | **54.27 ± 9.47** | **36.63 ± 6.79** | **0.004** |
| Anterior | **45.35 ± 12.05** | **35.46 ± 10.03** | **0.04** |
| Lateral | 62.01 ± 28.30 | 53.66 ± 30.08 | 0.49 |
| Posterior | 61.49 ± 29.22 | 44.13 ± 21.28 | 0.10 |
| Inferior | 46.64 ± 17.45 | 48.58 ± 19.64 | 0.47 |
| Septal | 56.19 ± 19.80 | 54.96 ± 28.31 | 0.91 |
| **Radial displacement (mm)** |  |  |  |
| Anterior Septal | 0.78 ± 0.10 | 0.68 ± 0.15 | 0.08 |
| Anterior | 0.68 ± 0.12 | 0.59 ± 0.24 | 0.31 |
| Lateral | 0.65 ± 0.10 | 0.69 ± 0.28 | 0.59 |
| Posterior | 0.75 ± 0.13 | 0.69 ± 0.19 | 0.36 |
| Inferior | 0.69 ± 0.15 | 0.65 ± 0.16 | 0.57 |
| Septal | 0.66 ± 0.07 | 0.67 ± 0.23 | 0.94 |
| **Circumferential displacement (degree)** | | | |
| Anterior Septal | 1.48 ± 0.80 | 1.01 ± 0.64 | 0.12 |
| Anterior | 1.37 ± 0.62 | 1.23 ± 0.63 | 0.58 |
| Lateral | 1.12 ± 0.42 | 0.89 ± 0.37 | 0.16 |
| Posterior | 1.28 ± 0.62 | 1.14 ± 0.91 | 0.67 |
| Inferior | 1.24 ± 0.54 | 1.05 ± 0.86 | 0.26 |
| Septal | 1.31 ± 0.55 | 0.94 ± 0.15 | 0.06 |

Data expressed as means ± SD. Significant values are represented in bold. CIA, collagen induced arthritis
